# Influence of In Situ Calcium Pectinate Coating on Metoprolol Tartrate Pellets for Controlled Release and Colon-Specific Drug Delivery

**DOI:** 10.3390/pharmaceutics14051061

**Published:** 2022-05-15

**Authors:** Pimphaka Wanasawas, Ampol Mitrevej, Nuttanan Sinchaipanid

**Affiliations:** Department of Manufacturing Pharmacy, Faculty of Pharmacy, Mahidol University, Bangkok 10400, Thailand; pimja1@yahoo.com (P.W.)

**Keywords:** in situ calcium pectinate, pellets, pectin, calcium chloride, alternate coating, fluidized bed

## Abstract

In situ calcium pectinate-coated pellets were proposed by applying an alternate coating method to drug-layered pellets to achieve colon-specific drug delivery. Solution layering of metroprolol tartrate, a water-soluble model drug, on inert core pellets was achieved using a centrifugal granulator followed by successive alternate coating with pectin and calcium chloride layers using a fluidized bed bottom spray coater. The effect of the coating sequence on the drug release was studied in phosphate buffer pH 7.4 and 6.0. These test conditions were used to mimic the physiological environments in the distal small intestine and proximal colon, respectively. The results showed that the in situ calcium pectinate layer was successfully generated from the alternate coating of pectin and calcium layers after hydration to form gelation, which was able to control the drug release. The coating sequence played an important role in the drug release. The outermost pectin layer tended to retard the drug release whilst the outermost calcium layer accelerated the release regardless of the number of coating layers. These findings indicate that the release behavior followed the Higuchi model, with the drug release from the coated pellets described by a diffusion control mechanism. It is concluded that the success of the in situ calcium pectinate-coated pellets in controlling the drug release is due to the coating of the outermost layer with pectin and the maintenance of the optimum ratio of calcium to pectin upon hydration.

## 1. Introduction

Pellets are small, free-flowing, and spherical particulates that are widely used in the pharmaceutical industry due to their flexibility regarding their dosage form design and development, ease of handling, reduced variation in GI transit time, and suitable shape for coating applications [1,2]. Pelletization can be achieved using many techniques, including extrusion-spheronization, melt/wet granulation, spray drying/congealing, and drug layering/coating. Among these processes, fluidized bed technology is a well-known process in pharmaceutical manufacturing, which can be tailored to a one-step operation for pellet production either by direct pelletization or drug layering on nonpariel sugar or microcrystalline cellulose beads.

Pectin is a natural product with long-established applications in the pharmaceutical industry. It is a polysaccharide polymer primarily consisting of galacturonic acid and its ester. Pectins are classified into high-methoxy and low-methoxy pectins based on the degree of methoxylation. Low-methoxy pectins are widely used in pharmaceutical applications due to their gelling ability via crosslinking with divalent cations, such as calcium [3,4]. Pectin and calcium pectinate polymers have been suggested for site-specific drug delivery targeting the colon as they have low water solubility and, at the same time, are easily and completely degraded by gut microflora [5]. Gamma scintigraphy studies have demonstrated that calcium pectinate capsules of 5-fluorouracil pass through the stomach and small intestine intact, and then release the drug inside the colon space [6,7]. Applications of calcium pectinate for colon targeting have been extensively formulated in many dosage forms, including pellets [8,9].

Our previous work [10] showed that the release behaviors of a slightly soluble drug, theophylline, from monolayer pectin and calcium pectinate film-coated pellets were significantly different and pH dependent. At a 10% coating level, the calcium pectinate film delayed drug release in a basic medium by up to 6 h compared to pectin film, which was about 3 h. However, monolayer calcium pectinate film with up to a 12% coating level did not retard the drug release of water-soluble drugs, such as metoprolol tartrate. The success of interfacial complexation coating has been reported for theophylline-coated pellets, in which the release was controlled by the pellet size, pectin type, and pectin concentration [11,12,13]. An alternate coating of pectin and calcium was applied, in which calcium pectinate gelation occurred after hydration of the coating, followed by crosslinking of pectin polymer with calcium ions at the interface [14], where diffusion-controlled interfacial complexation ensured an equal coating of all units and eliminated any coating defect due to irregular coating.

In this work, metoprolol tartrate was used as a model drug for water-soluble active compounds, and the application of in situ calcium pectinate-coated pellets was established with the alternate coating of pectin and calcium using fluidized bed technology for site-specific drug delivery targeting in the colon. The effects of the coating quantity and sequence of alternate coating on the drug release and release mechanism were studied.

## 2. Materials and Methods

### 2.1. Materials

Metoprolol tartrate (Karinco, Milano, Italy) was used as a model drug. Microcrystalline cellulose (MCC) seeds (Cellets^®^ 700), kindly provided by Pharmatrans Sanaq AG, Allschwil, Switzerland, were used as core materials. Hydroxypropyl methylcellulose (HPMC) (Methocel^®^ E15LV, Colorcon, Dartford, UK) was applied as a binder and sub-coating film layer. Pectin from citrus fruits (8.1% methoxy content) and calcium chloride dihydrate were supplied by Sigma (St. Louis, MO, USA) and Carlo Erba Reagenti (Cornaredo, Italy), respectively. Silicone emulsion (Dow Corning^®^ Antifoam 1614 DCS, AJAX Finechem, Sydney, Australia) was used as an antifoaming agent. Other excipients, including talcum, polyethylene glycol (PEG) 6000, and ethanol, were all of pharmaceutical or analytical grades.

### 2.2. Preparation of Metoprolol Tartrate Pellets

Metoprolol tartrate pellets were prepared using the solution layering technique. The formulation of the metoprolot tartrate solution is listed in Table 1. The drug was dissolved in a mixture of distilled water and ethanol at a ratio of 1:1 while HPMC solution was dispersed in a portion of hot water with the aid of a silicone emulsion and stirred to achieve a transparent solution without any lumps. After cooling, HPMC solution was mixed with metoprolol tartrate solution, talc was then added, and the resulting solution mixed homogeneously. Drug layering was prepared by a centrifugal granulator (Glatt^®^ GPCG-1, Glatt GmbH Process Technology, Binzen, Germany), equipped with a smooth disc. A 900-g batch of MCC seeds was loaded in the product chamber and the metoprolol tartrate preparation was sprayed onto the seeds through a 1.0 mm spray nozzle at a rate of 10–12 g/min with an atomization air pressure of 0.1 MPa to achieve 40% drug loading. The rotational disc speed was maintained at 250 rpm, the inlet temperature was set at 60 °C, and the fluidized air velocity was controlled at a rate of 80–85 m^3^/h. The product and outlet air temperatures were maintained in the range of 44–48 °C.

### 2.3. Preparation of Coated Metoprolol Tartrate Pellets

To smooth the metoprolol tartrate pellets prior to coating, sub-coating with HPMC was applied to a 5% weight gain and further coated with talcum to a 10% weight gain. Then, alternate coating with pectin (P) and calcium (Ca) was employed. The pectin solution consisted of 3% *w/w* pectin in water with glycerol as a plasticizer at 20% *w/w* of the pectin polymer. The calcium coating solution consisted of 4% *w/w* calcium chloride in water with HPMC as a binder at 4% *w/w* solution. The formulations of the sub-coating and coating are also summarized in Table 1.

The sub-coating and coating processes were carried out using a fluidized bed bottom spray coater (Glatt^®^ GPCG-1, Glatt GmbH Process Technology, Binzen, Germany). The operating conditions for each coating, i.e., sub-coating, pectin coating, and calcium coating, are described in Table 2. After the final coating, the coated pellets were dried for another 5 min in the chamber and then placed in a hot air oven at 50 °C for 4 h.

**Figure 1:**
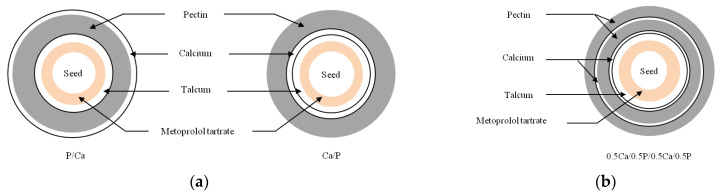
Illustration of alternate coated pellets with (**a**) coating sequence I, 2-layer coating; (**b**) coating sequence II, 4-layer coating based on the same coating quantity.

**Figure 2:**
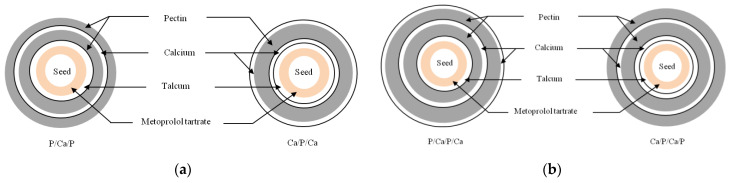
Illustration of alternate coated pellets with (**a**) coating sequence III, 3-layer coating; (**b**) coating sequence VI, 4-layer coating.

For the alternate coating, a 600-g batch was alternately sprayed with pectin and calcium solutions in 4 different coating sequences, i.e., a 2-layer coating was designed by alternate coating sub-coated pellets that were either initially coated with pectin and followed by calcium (P/Ca) or vice versa (Ca/P), defined as coating sequence I in Figure 1a. The coating sequence II was used to verify the drug release when the same coating quantity as coating sequence I was split into a 4-layer coating as 0.5Ca/0.5P/0.5Ca/0.5P as shown in Figure 1b. In the coating sequences III and IV, the alternate coating was assigned with an increasing coating quantity and different coating sequences are shown as 3-layer coating either as P/Ca/P or Ca/P/Ca in Figure 2a and 4-layer coating either as Ca/P/Ca/P or P/Ca/P/Ca in Figure 2b. Each pectin layer was set at a coating level of 20% weight gain and each calcium layer comprised 70 mg calcium per 1 g of pectin as reported in our previous study to result in the highest yield stress on the calcium pectinate film [10]. The monolayer coating with pectin film was set as the control formulation.

### 2.4. Evaluation of Coated Metoprolol Tartrate Pellets

#### 2.4.1. Morphology

The dried coated pellets were coated with gold under vacuum before micrographs were taken using a scanning electron microscope (SEM) (Jeol^®^, JSM-5410 LV, Jeol, Tokyo, Japan) to observe the shape and surface of the pellets.

#### 2.4.2. In Vitro Drug Release

The release profiles of core, sub-coated, and alternate-layer-coated pellets were investigated using the dissolution method for metoprolol tartrate tablets. The automated dissolution setup comprised a USP dissolution apparatus I (Model 72 RL, Hanson Research, Chatsworth, CA, USA) and a UV/VIS spectrophotometer (DU^®^ 650i, Beckman, Brea, CA, USA) equipped with 6 0.05-cm flow cells and a 6-channel peristaltic pump (Gilson^®^, Minipuls 3, Gilson, France). The dissolution media included 900 mL of simulated gastric fluid (SGF), and pH 6.0 and pH 7.4 phosphate buffer solutions maintained at 37 ± 0.5 °C. The amount of dissolved metoprolol tartrate was determined spectrophotometrically by measuring the maximum absorbance at 272 nm. The average amount of dissolved metoprolol tartrate was plotted against time. To explain the release kinetics of metoprolol tartrate from the alternate-layer-coated pellets, the obtained data were fitted in several mathematical models, i.e., zero order, first order, and Higuchi, using linear regression analysis and the coefficients of determination were determined.

## 3. Results and Discussion

### 3.1. Solution Layering of Metoprolol Tartrate Pellets

The solution layering technique was chosen to prepare metoprolol tartrate pellets. The addition of HPMC as a binder helped to strengthen pellet cracks or delamination from the core in the later stage of the coating processes. From our preliminary study, the fluidized bed bottom spray coater was primarily selected for the preparation; however, the pellets were found to stick together and adhere to the wall within a few minutes after the solution was sprayed. This is due to metoprolol tartrate, which is a hygroscopic compound under high humidity conditions [15]. The surface of the layered pellets was found to be tacky and adhered together, which impeded the flow of particles through the gap between the partition and orifice plate of the fluidized bed bottom spray chamber. With the centrifugal granulator, the tumbling action and high particle velocity helped generate rapid pellet turnover through the spray zone, allowing a higher liquid application rate and higher substrate layering capacity [16,17]. Using the aforementioned technique, the quality of the metoprolol tartrate pellets was demonstrated to be satisfactory for further coating.

### 3.2. Subcoating and Alternate Coating of Metoprolol Tartrate Pellets

**Figure 3:**
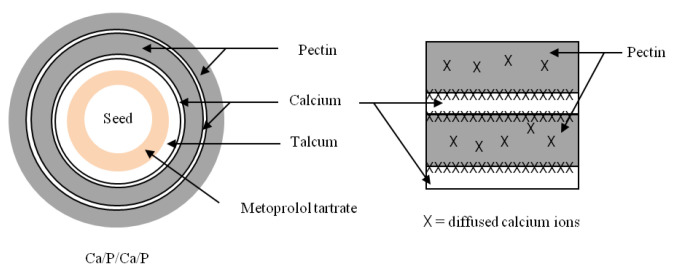
In situ calcium pectinate formation at the interface between the pectin and calcium layers.

Owing to the high degree of mechanical stress in the centrifugal granulator, the subsequent coating of metoprolol tartrate pellets was performed in a fluidized bed bottom spray coater to achieve uniform coating layers. During the coating process, each coating solution was atomized and sprayed into the fluidized bed concurrent with the fluidizing air. The coating droplets contacted with the drug-layered pellets in the spray zone, spread over the pellet surface, and coalesced together. Simultaneously, the coated pellets continued traveling upward and water was evaporated by the heated fluidizing air, which allowed dissolved materials to recrystallize and form solid bridges between the particles. The coating process continued until the desired amount of each layer in all coating sequences was obtained. Premature drug release in SGF within 30 min was observed in the pectin-coated pellets due to the high water solubility of metoprolol tartrate. Therefore, 10% *w*/*w* of talcum was additionally sub-coated onto the metoprolol tartrate pellets to increase the hydrophobicity of the pellets and avoid penetration of the dissolved drug into the hydrophilic film during the coating process [18]. Based on the ionic interaction between negatively charged carboxyl groups of pectin and positively charged calcium ions [8], a calcium pectinate coating layer was expected to form in situ. After exposure to the dissolution medium, calcium chloride was dissolved, providing calcium ions that then interacted with pectin molecules in the adjacent coating layer. Consequently, the in situ calcium pectinate layer gradually formed at the interface as represented in Figure 3. In comparison to conventional preparations of calcium pectinate using the dropping technique [9,19] or dipping method [13,20], the formation of an in situ calcium pectinate layer using the alternate spray coating technique has advantages as it does not require the removal process of excess calcium chloride solution and allows large-scale production with a continuous well-controlled process.

#### 3.2.1. Morphology of Coated Metoprolol Tartrate Pellets

**Figure 4:**
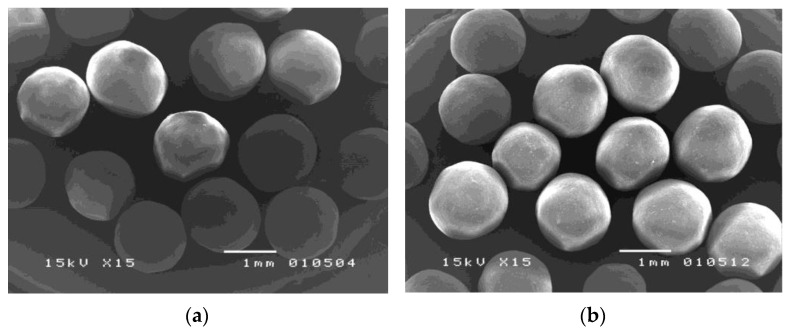
SEMs showing surface views at 15× magnification of (**a**) Ca/P/Ca/P-coated metoprolol tartrate pellets; (**b**) P/Ca/P/Ca-coated metoprolol tartrate pellets.

**Figure 5:**
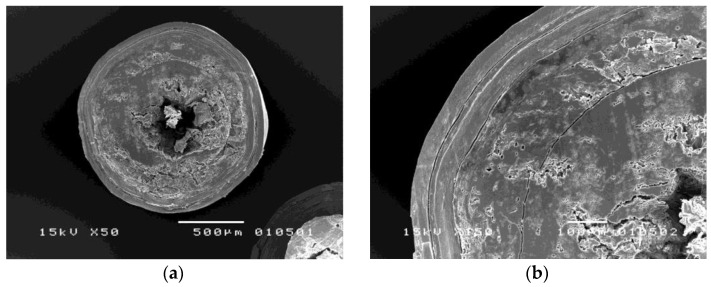
SEMs showing cross-sectional views at (**a**) 50× and (**b**) 150× magnification of the Ca/P/Ca/P-coated metoprolol tartrate pellets.

Figure 4 demonstrates that the different four-layer-coated metoprolol tartrate pellets exhibited a spherical shape with a smooth surface. The cross-sectional views of the Ca/P/Ca/P-coated pellets, as shown in Figure 5, show continuous film formation of each alternate layer. This indicates the good quality of the coating layer achieved with the use of the fluidized bed bottom spray coater. However, it is observed that the innermost calcium layer and the talcum layer combined with the drug layer. As the spray solutions of these three consecutive layers comprised the same ingredients, it is presumed that fusion might occur during over-coating of the former layer.

#### 3.2.2. In Vitro Drug Release

The dissolution profiles of the core and sub-coated metoprolol tartrate pellets were determined in SGF and phosphate buffer pH 6.0 and pH 7.4, showing complete dissolution within 10 min regardless of pH of the dissolution media (data not shown). This indicates that sub-coating did not affect the drug release in all dissolution media. With respect to the properties of calcium pectinate polymer, it has been reported that the polymer has low solubility and low swellability in acidic environments, and consequently contributes to negligible drug release [21]. In this study, therefore, the drug release from alternate-layer-coated metoprolol tartrate pellets was investigated and verified in pH 7.4 and pH 6.0 buffer solutions to mimic the conditions of the distal small intestine and proximal colon, respectively [8,9].

1. Effect of the inner and outer calcium layer of alternate layer coating

**Figure 6:**
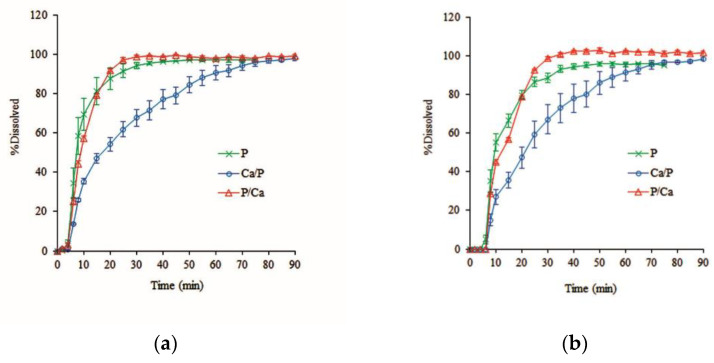
Effect of the inner and outer calcium layer on the dissolution profiles of alternate-layer-coated metoprolol tartrate pellets in phosphate buffer (**a**) pH 7.4; (**b**) pH 6.0 (mean ± SD, *n* = 3).

Figure 6 demonstrates that the Ca/P-coated pellets prolonged the drug release within 90 min compared to the rapid release shown by monolayer pectin-coated pellets, whereas P/Ca-coated pellets showed little delay in release during the first 10 min but showed rapid release after this time, which is similar to the monolayer pectin-coated pellets. All dissolution profiles showed a lag time of approximately 5 min for hydration and there was no difference in the dissolution profiles according to pH of the dissolution medium. The rapid drug release of P/Ca-coated pellets implies that after hydration, the outer calcium layer rapidly dissolves and releases calcium ions, which further interact with the inner pectin layer at the interface. However, a major portion of calcium ions might be dissolved in the dissolution medium, with only a little remaining at the interface. The lower concentration of calcium ions led to lower calcium pectinate reactions, resulting in the in situ calcium pectinate film having lower strength and being easily dissolved in the dissolution medium. On the other hand, the extended drug release from Ca/P-coated pellets showed that after hydration, the outer pectin layer swelled and acted as a barrier to impede the dissolution of calcium ions from the inner calcium layer. This provided enough time for the interaction between pectin molecules and calcium ions to form the in situ calcium pectinate film.

2. Effect of the number of layers of alternate layer coating

**Figure 7:**
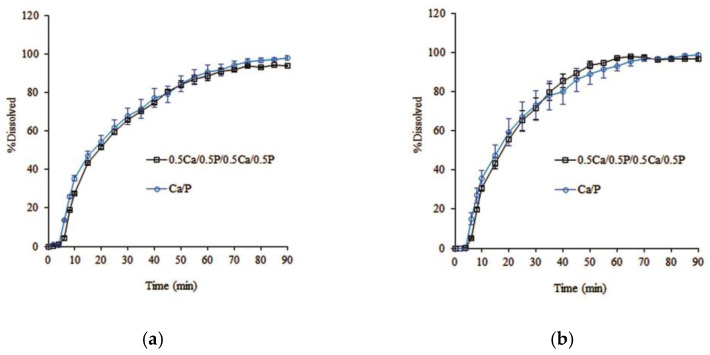
Effect of the number of layers on the dissolution profiles of alternate-layer-coated metoprolol tartrate pellets in phosphate buffer (**a**) pH 7.4; (**b**) pH 6.0 (mean ± SD; *n* = 3).

The influence of the number of alternate layers on the dissolution profiles of the coated metoprolol tartrate pellets with the same coating quantity was studied in Ca/P-coated pellets. After applying an equal amount of pectin and calcium coating, the alternate 4-layer coating with 0.5Ca/0.5P/0.5Ca/0.5P was designed to improve the distribution of calcium ions in the pectin layers to consequently enhance the gelation of calcium pectinate after hydration. However, the drug release from the alternate four-layer-coated pellets was similar to the alternate two-layer-coated pellets, as shown in Figure 7.

No effect of pH on drug release was observed. This result indicates that the calcium layer, either in the alternate 2-layer or 4-layer coatings, completely dissolved and released calcium ions, which then rapidly penetrated the pectin network to effectively form the in situ calcium pectinate film. This drug release is attributed to the coating quantity, which was also included in the present study.

3. Effect of coating quantity and sequence of alternate layer coating

**Figure 8:**
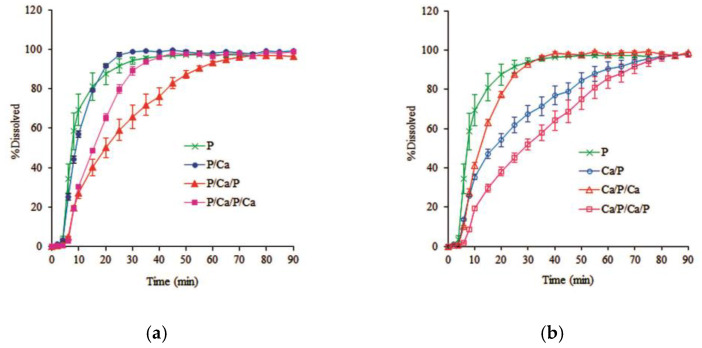
Effect of the coating quantity and sequence on the dissolution profiles of alternate layer coated metoprolol tartrate pellets in pH 7.4 phosphate buffer solution: (**a**) initially coated with a pectin layer; (**b**) initially coated with a calcium layer (mean ± SD; *n* = 3).

**Figure 9:**
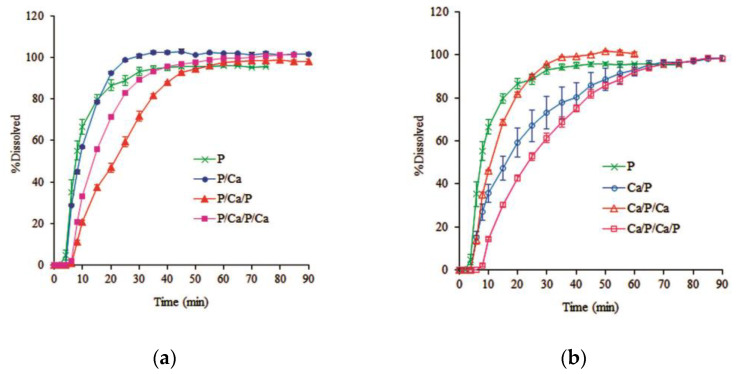
Effect of the coating quantity and sequence on the dissolution profiles of alternate layer coated metoprolol tartrate pellets in pH 6.0 phosphate buffer solution: (**a**) initially coated with a pectin layer; (**b**) initially coated with a calcium layer (mean ± SD; *n* = 3).

The alternate three-layer and four-layer coatings were applied. The drug release was also compared to the alternate two-layer and monolayer pectin-coated pellets. The dissolution profiles of these coated pellets in phosphate buffer pH 7.4 and pH 6.0 are represented in Figure 8 and Figure 9, respectively. The lag time was slightly longer when the coating quantity was increased, and the effect of the dissolution pH was also noticeable. The drug release in pH 6.0 was slightly faster than in pH 7.4. These results agree with a report indicating that pectin molecules are almost completely ionized at pH 6.0 [22].

The increased coating quantity with the equal pectin layers evidently extended the drug release; however, the coating sequence also had an impact on the drug release behavior. The release rates of metoprolol tartrate from the pellets with the innermost pectin coating were in the following order: P > P/Ca > P/Ca/P/Ca > P/Ca/P as shown in Figure 8a and Figure 9a. It is interesting to note that the 4-layer coating, P/Ca/P/Ca, provided faster drug release than the 3-layer coating, P/Ca/P, conforming with the release behavior shown by the alternate 2-layer coating, where the outer calcium layer accelerated the drug release. Regarding the innermost calcium coating, Figure 8b and Figure 9b show that the release rates were in the following order: P > Ca/P/Ca > Ca/P > Ca/P/Ca/P. The drug release of the 3-layer coating, Ca/P/Ca, was obviously more rapid than that of the 2-layer coating, Ca/P. This result corresponded with our previous work showing that an increasing in the calcium to pectin ratio reduced the yield stress on the calcium pectinate film [10].

4. Mathematical models of alternate-layer-coated pellets

Several mathematical models were used to explain the release kinetics of metoprolol tartrate from the alternate-layer-coated pellets, including the zero order, first order, and Higuchi model. Table 3 shows that the Higuchi model showed good coefficients of determination for the drug release under the pH 7.4 and pH 6.0 conditions, indicating that the drug release followed a diffusion control mechanism. This finding is in agreement with our previous study, which showed that diffusion control played a role in the drug release from a monolayer of pectin- and calcium pectinate-coated pellets [10]. Based on the diffusion control release kinetic, it can be concluded that the in situ calcium pectinate layer in this study successfully formed after hydration and the dissolved drug diffused through the gel layer of the coating layer. The thicker gel layer at the higher coating quantity caused slower drug diffusion in the reservoir, resulting in the formation of a depletion layer adjacent to the gel surface according to the Higuchi model [23], regardless of the number of coating layers.

## 4. Conclusions

The highest ability to control drug release was observed in the alternate four-layer coating, Ca/P/Ca/P, where in situ interfacial complexation possibly took place at the highest level in relation to the increased coating quantity. On the contrary, the outermost calcium layer apparently accelerated the drug release regardless of the number of layers. It was observed that in the case of the 4-layer coating, P/Ca/P/Ca, it provided a faster drug release than the 3-layer coating, P/Ca/P, where pectin was applied in the same quantity. This result indicates that the coating sequence of the calcium layer affected the in situ crosslinking system. Calcium ions in the outermost layer led to weakness in the calcium pectinate coating layer. Furthermore, the effect of the calcium amount was observed in the coating sequence of P/Ca/P and Ca/P, where the outermost layer was the pectin coating. The drug release from both sequences was similar in pH 7.4 but different in pH 6.0 phosphate buffer. The P/Ca/P-coated pellets provided faster drug release in pH 6.0 phosphate buffer than the Ca/P-coated pellets, although the amount of pectin was higher. This can be explained by the almost complete ionization of pectin at pH 6.0. The lower ratio of calcium to pectin in the P/Ca/P coating may have resulted in the lower strength of the calcium pectinate film and thus provided higher drug release. This result indicates that the success of the in situ calcium pectinate coating layer depended on the outermost coating of the pectin layer and maintenance of the ratio of calcium to pectin in the multiple-layer-coated pellets upon hydration.

## Figures and Tables

**Table 1:** Formulations of the metoprolot tartrate solution, sub-coating, pectin coating (P), and calcium coating (Ca).

Ingredients	% *w/w*			
	Drug Layering	Sub-Coating	Coating	
			Pectin (P)	Calcium (Ca)
Metoprolol tartrate	30.0	-	-	-
HPMC E15 LV	3.0	4.0	-	4.0
Pectin	-	-	3.0	-
Calcium chloride	-	-	-	4.0
PEG 6000	-	0.8	-	0.8
Talcum	2.0	0.8	-	0.8
Glycerol	-	-	0.6	-
Silicone emulsion, 10%	0.2	0.2	-	0.2
Ethanol and water (1:1) qs	100	-	-	-
Water qs	-	100.0	100.0	100.0

**Table 2:** Operating conditions for sub-coating, pectin coating, and calcium coating.

Parameters	Operating Conditions		
	Sub-Coating	Pectin Coating	Calcium Coating
Inlet temperature, °C	50	65	50
Fluidized air velocity, m^3^/h	75	75	75
Product and outlet temperature, °C	32–34	35–38	32–34
Atomization pressure, MPa	0.25	0.25	0.25
Spray rate, g/min	10–12	15–18	10–12

**Table 3:** Coefficients of determination obtained from linear regression analyses of various mathematical models.

pH	Coating Sequence	Zero Order Q_t_ = K_0_t + Q_0_	First Order log Q_t_ = K_1_t + log Q_0_	Higuchi Q_t_ = K_H_t^1^^/2^
7.4	P	0.8190	0.7128	0.8734
	P/Ca	0.9663	0.8732	0.9864
	P/Ca/P	0.9582	0.8458	0.9929
	P/Ca/P/Ca	0.9826	0.9009	0.9974
	Ca/P	0.9154	0.7374	0.9725
	Ca/P/Ca	0.9468	0.7616	0.9799
	Ca/P/Ca/P	0.9867	0.9009	0.9992
6.0	P	0.8225	0.7270	0.8845
	P/Ca	0.9779	0.9076	0.9934
	P/Ca/P	0.9840	0.8567	0.9974
	P/Ca/P/Ca	0.9517	0.8584	0.9819
	Ca/P	0.9152	0.7647	0.9714
	Ca/P/Ca	0.9269	0.7540	0.9663
	Ca/P/Ca/P	0.9517	0.8101	0.9898

Q_0_ and Q_t_ represent the amount of drug release at initial and time t, respectively. K_0_ is the zero order release constant (%min^−1^), K_1_ is the first order release constant (min^−1^), and K_H_ is the Higuchi dissolution constant (%min^−1/2^).

## Data Availability

Not applicable.
